# PEEK versus Silicon Interspinous Spacer for Reduction of Supradjacent Segment Degeneration following Decompression and Short-Segment Instrumentation for Degenerative Lumbar Spinal Stenosis

**DOI:** 10.1155/2018/1623647

**Published:** 2018-08-08

**Authors:** Panagiotis Korovessis, Vasileios Syrimpeis, Vasileios Tsekouras, Konstantinos Vardakastanis, Peter Fennema

**Affiliations:** ^1^Orthopaedics Department, General Hospital of Patras, Tsertidou Str 1, 26224, Greece; ^2^AMR Advanced Medical Research GmbH, Hofenstrasse 89b, CH-8708 Männedorf, Switzerland

## Abstract

**Purpose:**

A retrospective study that aims to report Adjacent Segment Degeneration (ASD) incidence and spinopelvic balance in short lumbosacral instrumentation for degenerative lumbar spinal stenosis. Although ASD is a common complication following lumbar fusion, the effect of an interspinous spacer (IS) in the supradjacent segment in short lumbosacral instrumented fusion and its interaction with spinopelvic balance has not been studied adequately.

**Methods:**

From 55 consecutive age-, diagnosis-, and gender-matched patients aged 60±11 years, 17 (Group R) received PEEK IS; 18 (Group S) received Silicon IS compared with 20 controls (Group C) without receiving any IS. The functional outcome was evaluated with VAS and ODI. Spinopelvic balance was evaluated using SVA, T_12_-S_1_ LL, SS, PT, PI, and supradjacent segment disc heights. All spines were preoperatively balanced (SVA<40 mm).

**Results:**

The follow-up averaged out to 56±11 months. VAS and ODI scores improved postoperatively in all 3 groups. SS and anterior disc height in the supradjacent free segment increased postoperatively compensatory to spinal alterations. Although 6, 4, and 5 patients from Groups R, S, and C, respectively, showed radiological progression of the preoperative degeneration grade in the supradjacent disc, only 2, 1, and 2 patients in Groups R, S, and C, respectively, developed symptomatic ASD in the 1^st^ supradjacent segment solely. No additional surgery was required in any patient.

**Conclusion:**

ASD incidence in the supradjacent segment following short lumbar fusion did not statistically significantly differ between PEEK and Silicon IS. There was a trend towards lower ASD incidence in Silicon IS. IS reduced ASD in both 1^st^ and 2^nd^ supradjacent segments. The authors speculate that soft stabilization provided by IS may be more advantageous for preventing ASD. This trial is registered with* ClinicalTrials.gov*NCT03477955.

## 1. Introduction

Decompression and instrumented fusion is the most commonly performed procedure for the treatment of degenerative lumbar spinal stenosis (DLSS) [1, 2]. Instrumented spinal fusion increases significantly motion and forces acting mainly on the supradjacent segment of the instrumented lumbar area [3] resulting in ASD and less commonly in symptomatic adjacent segment disease [4].

ASD incidence ranges from 0 to 100% [5] while the reported risk for symptomatic ASD per year ranged from 0.6 to 3.9% [6]. ASD in DLSS occurs most frequently (89%) in the supradjacent segment of instrumented fusion, while it is rare (3.7%) in the subjacent segment [7]. Among the reported risk factors for ASD following spinal fusion, age, preexisting segment degeneration, and sagittal imbalance have been included [5, 6, 8].

Although sagittal balance restoration is important in patients undergoing deformity surgery [9], sagittal balance restoration is less emphasized in patients with DLSS. Old age, PI-LL mismatches, high PT, and high disability index scores were associated with sagittal imbalance [10, 11]. There exists an interaction between sagittal imbalance and ASD, while both are associated with poor health quality and pain [10, 12, 13].

Although there is no recommendation, there are clinical studies that reported successful use of IS to prevent ASD following lumbar spine fusion [8, 14–16].

Based on previous clinical studies [5, 14–16] the authors hypothesized that by adding an IS at the supradjacent segment, above a rigid short lumbosacral instrumentation, in a balanced spine, a decreased incidence of ASD may occur.

The* primary outcome measure* of this study was the ASD incidence in the spines that received PEEK IS versus those that received Silicon IS; and the* secondary outcome measure* was to correlate potential spinopelvic alignment changes postoperatively with ASD.

## 2. Patients and Methods

This study included initially two consecutive selected groups (R and S) of 20, gender-, age-, and diagnosis-matched adult patients each. The patients underwent a primary wide decompression plus 1- or 2 segment pedicle screw fixation with posterolateral instrumented fusion with the addition of PLIF in one segment or no PLIF (Table 1), between April 2006 and November 2009 for symptomatic DLSS. No concomitant decompression in the adjacent segment was performed in any patient of the three groups because the degeneration grades were low and there were no stenosis symptoms from the adjacent segment. The* inclusion criteria *were preoperative MRI with degeneration grades ≤III [17] at the 1^st^ supradjacent lumbar segment. The patients were randomly selected to receive one of two IS: Wallis IS (Zimmer, Inc., San Clemente, CA, USA) (Group R) or the DIAM IS (Medtronic Inc., Sunnyvale, CA, USA) (Group S). Twenty individuals from our historical series (Group C), who had received by the first author the same surgery without the addition of IS and similar degeneration grade in the adjacent segments (two above and one below fusion), were selected to fit diagnosis, age, functional, and radiological parameters (Table 2). The insertion of IS was performed according to the manufactures' instructions, with meticulous preservation of the capsule and facet joints in the unfused segments to reduce iatrogenic ASD.

The* exclusion criteria* were body mass index ≥40kg/m^2^, severe osteoporosis, lumbar fracture, preoperative SVA>4 cm, spondylolisthesis grades ≥II or spondylolytic lesion, and acquired spinous process insufficiency in the supradjacent segment cephalad to instrumentation. All patients had moderate to severe lower back pain and/or numbness in the lower extremity/-ies before surgery and had received for a minimum of 6 months conservative therapy without relief. Patients were assessed with the ODI score and the lower limb pain plus the back pain magnitude with the VAS (0–10 scale). This clinical trial was approved by the institutional ethical committee and informed consent was obtained from all individual participants.

The preoperative and postoperative roentgenographic work-up included the following: (a) standing whole spine anteroposterior digital roentgenogram, (b) lateral digital roentgenograms, (c) sitting lateral dynamic (flexion/extension), and (d) supine oblique views for spinal fusion estimation.

**Figure 1:**
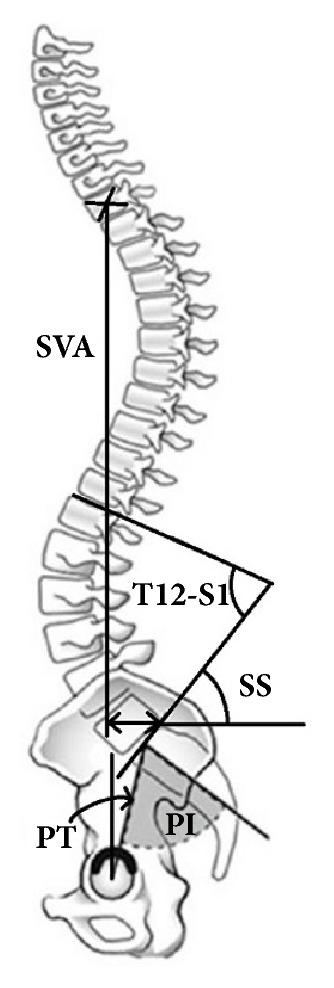
The radiological parameters PI, SS, PT, LL (T_12_-S_1_), SVA, and SL.

**Figure 2:**
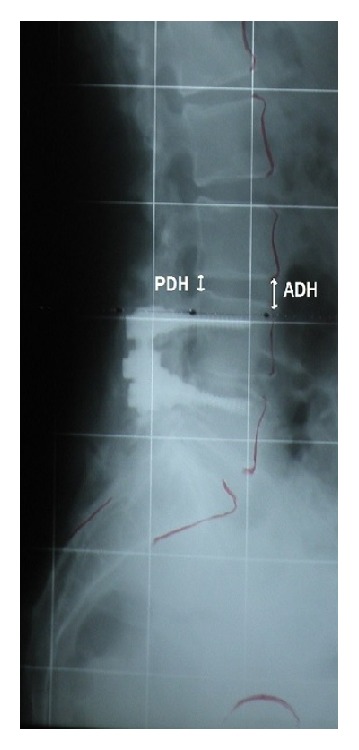
Anterior disc height ratio (ADHr) and posterior disc height ratio (PDHr).

Pelvic Incidence (PI) (the angle between the line perpendicular to the sacral plate at its midpoint and the line connecting this point to the femoral heads axis), Sacral Slope (SS) (the angle between the horizontal and the sacral plate), Pelvic Tilt (PT) (the angle created by a line running from the sacral end plate midpoint to the center of the bifemoral heads and the vertical axis), T_12_–S_1_ Lordosis (LL) (the angle formed by the lines drawn at the upper endplate of T_12_-vertebra and upper endplate of S_1_), and SVA (the distance from the posterior S_1_ endplate edge to the T_1_-plumbline) were measured (Figure 1). In addition, Segmental Lordosis (SL) (the angle formed between the upper and lower endplates at the supradjacent unfused segment), anterior disc height (ADH), and posterior disc height (PDH) were measured (Figure 2). Sagittal imbalance according to Schwab et al. is defined when SVA≥40 mm [13]. Spinal fusion was radiologically evaluated 8-12 months postoperatively using the Christensen grading [18].

**Figure 3:**
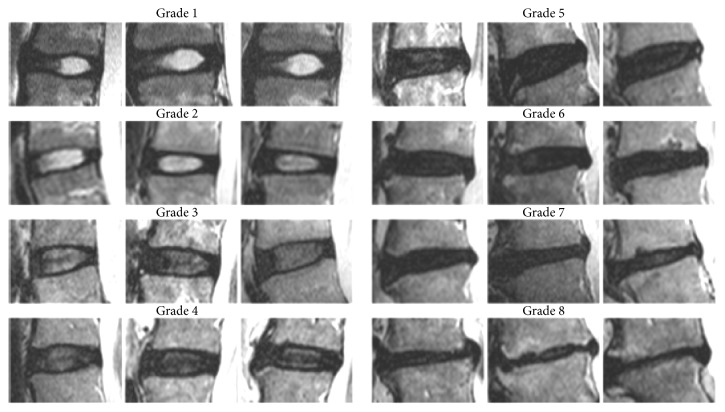
MRI images of the Modified Grading System for Lumbar Disc Degeneration. This material is used after author's permission [17].

The modified Pfirrmann [17] MRI classification (Table 3 and Figure 3) was used both preoperatively and 3-5 years postoperatively for disc degeneration grading in the 1^st^ and 2^nd^ supradjacent and subjacent segment (Tables 4 and 5). ASD with clinical significance was defined as the worsening of low back pain despite solid fusion and the absence of any surgery-related complication. Facet joint injection with local anesthetic under image intensifier was made for identification and localization of the pain source.

Sitting lateral dynamic X-rays (flexion/extension) and supine oblique views for spinal fusion determination were used in all 55 patients. CT scan was used for spinal determination of the posterolateral and PLIF spinal fusion rate.

The reliability (intra- and interrater) of the disc degeneration grade and spinal fusion in each particular spine was tested by two independent unbiased observers: one senior orthopedic radiologist and one experienced spine surgeon using the kappa value. Difference in grading more than one degree was regulated by consensus.

Subjects of Groups R, S, and C were followed through discharge for 3 to 5 years. Functional and radiological examination results were recorded for this study 6 and 24 months and thereafter once every 2 years after index surgery and until the final observation (Tables 6 and 7).

### 2.1. Interspinous Spacers

**Figure 4:**
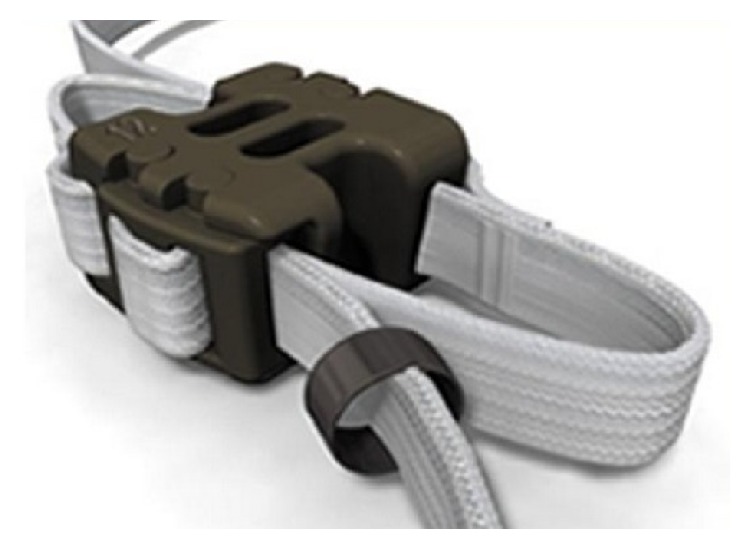
The second-generation Wallis interspinous spacer.

Second-generation Wallis (Figure 4) is a floating system, consisting of a PEEK (polyetheretherketone) block that is considered in this study compared to DIAM rigid. It is augmented by two woven Dacron ribbons, which are wrapped around the spinous processes and fixed under tension.

**Figure 5:**
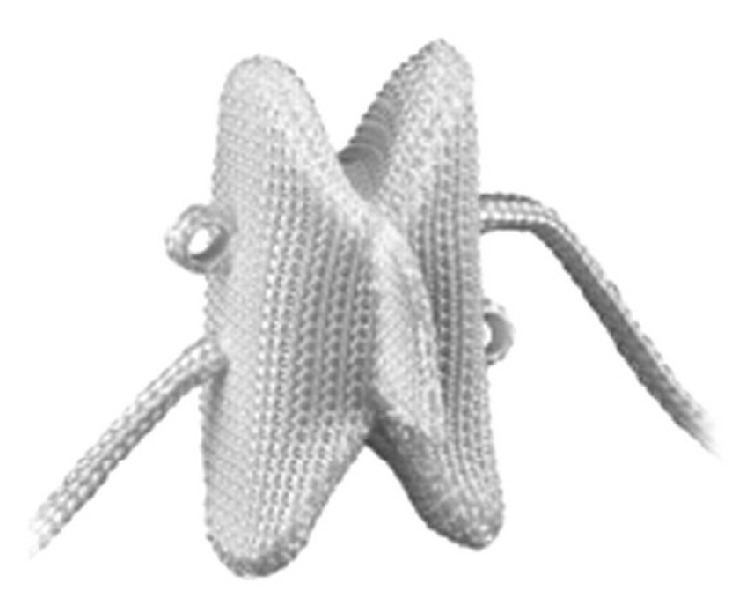
The DIAM interspinous spacer.

DIAM (Figure 5) is a silicon core covered by a polyester sleeve. It is held in position by three mesh bands, two around each spinous process and one around the supraspinous ligament.

DIAM's and Wallis' potential benefits are their alternative to spinal fusion, their fit between the interspinous processes, and their function as a shock absorber that reduces loads from the surrounding vertebrae [3, 14, 19, 20].

### 2.2. Statistical Data Analysis

Data were analyzed using the SPSS Software (v.18, SPSS, Inc., Chicago, IL, USA). Continuous data were reported as mean±SD.

Global and individual group statistical analysis was performed using the Fisher exact test for categorical variables.

Simple linear regression analysis was used to compare different parameters.

Two-sided p-values<0.05 were considered statistically significant.

Clinical success was defined as a ≥20 percentage point improvement in pain VAS score and a ≥15 percentage point improvement in ODI.

The kappa value was used for inter- and intrarater agreement in the evaluation of spinal fusion and Pfirrmann degeneration grade.

The research that is reported in the manuscript has been performed with the approval of the GHP's Ethics Committee and was carried out in compliance with the Helsinki Declaration.

## 3. Results

From the 60 patients who were initially enrolled in this study, 55 patients were finally separated in three groups: 17 patients (Group R); 18 patients (Group S); and 20 patients (Group C); they were finally available for the latest evaluation after they have completed a minimum of 3 years of follow-up after index surgery.

The female/male ratio per group was as follows: Group R 10:7; Group S 12:6; and Group C 13:7. The age of the patients in Groups R, S, and C were 64±17, 59±13, and 61±12 years, respectively, (p=0.49) (Table 8).

The L5-S1 segment was included in the fusion in two, four, and three patients in each of the Groups R, S, and C, respectively (Table 1). Whenever the L5-S1 segment was included in the instrumentation, it was reinforced with a PLIF.

TLIF received 14/17 patients in Group R, 13/18 in Group S, and 15/20 in Group C (Table 1). A single TLIF was used in the patients who received circumferential fusion.

The inter- and intrarater kappa-values for Pfirrmann degeneration grading pre- to postoperatively at the1^st^ and 2^nd^ supradjacent and subjacent segments ranged from 0.82 to 0.91. The inter- and intrarater kappa-values for Christensen spinal fusion evaluation ranged from 0.93 to 0.97.

The mean follow-up was 56±11 months, range 37 to 70 months, and did not differ between Groups R, S, and C (Fisher, p=0.24).

For the 5 patients, who were not available for the final observation (>3 years) for different reasons (residence change, death due to unrelated reasons) and were excluded from the analysis, in the reviewed charts, no complications or other surgery-related events or revision surgery had been recorded until the latest observation.

### 3.1. Functional Results

All 3 groups showed statistically significant improvements in all clinical outcomes six months postoperatively, without further significant changes until the final observation. No significant differences between the groups in the changes of functional scores (VAS, ODI) were shown (Table 6).

### 3.2. Roentgenographic/MRI Results

Preoperatively, disc degeneration grade increases with age (P<0.01).

SS increased significantly (P=0.015) in the 6-month postoperative evaluation in Group R, while it increased marginally statistically significantly (P=0.054) in Group C.

ADH at the IS insertion segment increased significantly (P=0.039) in Group R and marginally significantly (P=0.06) in Group C, 2 years postoperatively until the final observation (Table 7).

### 3.3. Complications

#### 3.3.1. Surgical

Accidental dural tears occurred and were sutured in the same session in two patients (one in each of the Groups S and C). Deep hematomas occurred in the first week postoperatively in one patient in Group C and in one patient in Group S and were evacuated successfully.

Urinary tract infection occurred in two patients (one in Group R and one in Group C).

Pneumonia turned up in one patient in Group S and was treated with antibiotics.

No intraoperative spinous processes fracture occurred during PEEK or Silicon IS implantation.

#### 3.3.2. Radiological

In 6, 4, and 5 patients of Group R, Group S, and Group C, respectively, there was a progression of Pfirrmann degeneration grade at the 1^st^ IS insertion segment (Table 4). In the 2^nd^ supradjacent and subjacent segments, ASD grade remained postoperatively<III grades (Tables 4 and 5).

*Adjacent Segment Disease*. Only 2 patients (12%) in Group R, 1 patient (5%) in Group S, and 2 patients (10%) in Group C (P=0.43) developed adjacent segment disease with pain localized solely in the 1^st^ supradjacent segment. There was no patient operated on for adjacent segment disease (Table 4).

## 4. Discussion

Regarding the first outcome measure, ASD incidence in the supradjacent segment was similar in Groups R and S. Regarding the second outcome measure, there were no significant changes in sagittal spinopelvic alignment in any group.

However, in this study there was a trend that, in the spines that received Silicon IS, the symptomatic ASD was lower (5%) compared to PEEK (12%) and controls (10%). This is in compliance with the previously reported results with transpedicular instrumentation (12.2–18.5%) [21]. Consistently, with previous papers, no patient developed symptomatic ASD in the subjacent and 2^nd^ supradjacent segment [22, 23]. In a previous similar study [14], the incidence of ASD both in the 1^st^ and 2^nd^ cephalad supradjacent unfused segment, following transpedicular fixation without IS, was higher (28.6%), than in the PEEK (Wallis) group (4.1%). Previous studies [14, 21] showed that the addition of Wallis protected both supradjacent segments from ASD and this was shown in our study with PEEK and Silicon IS. Postoperatively there was an increase of degeneration grade in the supradjacent segment only in the spines with grade III disc degeneration, justifying a previous study [24].

Sagittal spinal imbalance with impact on ASD may occur following instrumented lumbar fusion, because spinal fusion alters the biomechanics of the spine. Subsequently, the loss of motion at the fused levels is at least theoretically compensated by changes of sagittal spinopelvic balance and increased motion in the unfused segments [25]. Among the different spinopelvic parameters that were studied in this paper, there was only a postoperative increase of SS that represented an essential compensatory mechanism to resist the reduction of the anteversion of the pelvis, even in preoperatively balanced spines [16]. Furthermore, the increase of anterior disc height in the spines of Groups R and C may indicate adaptation in order to maintain the preoperative sagittal balance.

Spinal fusion is considered the gold standard for treatment of spinal degenerative disease, although there are several complications associated with this technique, such as nonunion, instrumentation failure, infection, and pain in the donor area if an iliac bone graft is used. Moreover, an increased range of movement at adjacent segments after spinal fusion may increase the risk of ASD. To avoid some of these undesirable effects, dynamic stabilization systems and motion-preserving surgeries (disc replacement, Graff ligamentoplasty, and Dynesys) are increasingly gaining interest in order to avoid the adverse effects of spinal fusion [26].

Wallis and DIAM represent newer advents of dynamic stabilization for the unfused lumbar spine similarly with that provided by Graff ligaments and Dynesys elastic stabilization. A recent clinical study that compared Dynesys stabilization with PLIF fixation in the lumbar degenerative disease showed that Dynesys stabilization partially preserves the range of motion of the stabilized segments, limits hypermobility in the upper adjacent not-fixed segment, and may prevent the occurrence of ASD [27].

In the relative literature there are contradictory results regarding the usefulness of IS in reducing ASD in DLSS [14, 19, 20]. IS related complications such as recurrent lumbar disc herniation, bone resorption, spinous process fractures, and dural tear at the level of IS insertion have been reported [20]. In this series there were two cases of dural tear that were successfully sutured. The absence of spinous process fractures and osteolysis may be due to the lack of significant osteoporosis in our patients.

Few temporary neurologic complications were observed in this series mostly related to PLIF insertion and nerve root retraction as in other similar studies also reported with Dynesys dynamic stabilization and PLIF [27].

The* strengths of the study* are as follows: (a) an interesting hypothesis that the use of an IS in the most commonly degenerated segment, which is supradjacent to rigid lumbar fusion, may reduce symptomatic ASD; (b) while all three groups were selected consecutively, the patients were treated by the same one experienced surgeon; (c) there was a meticulous assessment of specified radiographic and functional parameters; and (d) prespecified inclusion and exclusion criteria were established.

The* limitations of this study* are as follows: (a) the small number of patients in each group; (b) the retrospective design of the study; and (c) the lack of power analysis preoperatively.

In conclusion, the present study found that short lumbosacral fixation in balanced spines does not significantly alter the preoperative spinopelvic alignment. No significant differences in symptomatic ASD incidence were found between PEEK, Silicon IS, and groups without IS, but there was a trend towards lower ASD in the Silicon Group. Further prospective studies with a greater number of patients and longer follow-up, focusing on clinical and radiographical outcomes, are warranted.

## Figures and Tables

**Table 1:** Patients with circumferential fusion presented per group and level of PLIF insertion.

**Group**	**Segments fused**			
	*L4/L5*	*L5/S1*	*No PLIF*	Total
**R**	12	2	3	**17**

**S**	9	4	5	**18**

**C**	12	3	5	**20**

Total	**33**	**9**	**13**	**55**

**Table 2:** Baseline radiographic parameters comparison (P-values, unpaired t-test).

**Group**	**T12-S1 (LL)**	**SVA**	**SS**	**PI**	**PT**	**SL**	**ADH**	**PDH**

**Wallis vs DIAM**	0.74	0.55	0.6	0.73	0.37	0.21	0.0005	0.023

**DIAM vs Control**	0.16	0.14	0.16	0.06	0.09	0.008	0.008	0.024

**Control vs Wallis**	0.08	0.06	0.32	0.06	0.7	0.68	0.68	0.009

**Table 3:** The Modified Grading System for Lumbar Disc Degeneration*∗*. This material is used after author's permission [17].

Grade	Signal from Nucleus and Inner Fibers of Annulus	Distinction Between Inner and Outer Fibers of Annulus at Posterior Aspect of Disc	Height of Disc
1	Uniformly hyperintense, equal to CSF	Distinct	Normal
2	Hyperintense (>presacral fat and <CSF)±hypointense intranuclear cleft	Distinct	Normal
3	Hyperintense though < presacral fat	Distinct	Normal
4	Mildly hyperintense (slightly > outer fibers of annulus)	Indistinct	Normal
5	Hypointense (=outer fibers of annulus)	Indistinct	Normal
6	Hypointense	Indistinct	<30% reduction in disc height
7	Hypointense	Indistinct	30%-60% reduction in disc height
8	Hypointense	Indistinct	>60% reduction in disc height

*∗*Grades 1, 2, and 3 are based on the signal intensity of the nucleus and inner fibers of annulus. For grade 4, the margins between the inner and other fibers of the annulus at the posterior margin of the disc are indistinct. For grade 5, the disc is uniformly hypointense, although there is no loss of disc space height. For grades 6, 7, and 8, there is progressive loss of disc space height. These could be broadly classified as mild and moderate to severe loss of disc space height. Very occasionally, although obvious disc collapse is present, hyperintense signal from the nucleus and inner fibers of the annulus is preserved. This is referred to by a double entry, e.g., 4/7 with the former reporting the disc signal and the latter reporting the degree of collapse.

**Table 4:** Pfirrmann degeneration grade in MRI in the 1^st^ supradjacent segment preoperatively till the final observation.

**Group**	**Preoperatively**		**Postoperatively (>3 years postop)**			
	**II**	**III**	**II**	**III**	**IV**	**V**
**R (n=17)**	***8***	***9***	8	7	0	**2** **∗**

**S (n= 18)**	***7***	***11***	7	9	1	**1** **∗**

**C (n=20)**	***8***	***12***	8	8	2	**2** **∗**

*∗*Cases with symptomatic ASD.

**Table 5:** Pfirrmann classification in the 2^nd^ supradjacent and subjacent segment preoperatively till the final follow-up.

**2nd Supradjacent Group**	**Preoperatively**		**Postoperatively**		
	**II**	**III**	**I**	**II**	**III**
**R (n=17)**	***16***	***1***	15	2	0

**S (n= 18)**	***17***	***1***	16	2	0

**C (n=20)**	***18***	***2***	17	3	0

**Subjacent Group**	**I**	**II**	**I**	**II**	**III**

**R (n=14/17)** *∗∗*	11	3	10	4	0

**S (n= 16/18)** **∗**	12	4	11	3	2

**C (n=17/20)** **∗** **∗** **∗**	14	3	13	3	1

*∗*,*∗∗*,*∗∗∗* The segments per group with L5-S1 fusion that were excluded from ASD evaluation.

**Table 6:** Functional outcomes of 55 patients who received short lumbosacral fusion.

**Group**	**ODI**						**VAS-Leg**						**VAS-Back**					
	**Preop**	**Post Op**	**pre/post**	**Post Op-2**	**F-up**	**Post-2/F-up**	**Preop**	**Post Op**	**pre/post**	**Post Op-2**	**F-up**	**Post-2/F-up**	**Preop**	**Post Op**	**pre/post**	**Post Op-2**	**F-up**	**Post-2/F-up**
	**ODI**	**6 months**	**P-value**	**2 Years**	**max**	**P-value**	**VAS**	**6 months**	**P-value**	**2 Years**	**max**	**P-value**	**VAS**	**6 months**	**P-value**	**2 Years**	**max**	**P-value**
R	37±12	18±16%	**0.001**	19±14%;	20±15%;	0.56	5.9 ± 1.1	2.3 ± 0.5	**0.001**	2.4 ± 0.4	2.6 ± 0.5	0.56	4.3 ± 0.4	1.5 ± 0.3	**0.001**	1.6 ± 0.4	1.6 ± 0.3	0.91
S	39±12%	20±16%	**0.001**	21±15%	20±12%	0.84	5.5 ± 0.5	2.1 ± 0.4	**0.001**	2.5 ± 0.6	2.7 ± 0.5	0.65	4.7 ± 1.1	2.0 ± 0.1	**0.001**	2.2 ± 021	2.3 ± 0.2	0.67
C	38±12%	19±14%.	**0.001**	20±13%.	21±13%.	0.91	5.7 ± 0.3	2.2 ± 0.2	**0.001**	2.6 ± 0.3	2.6 ± 0.5	0.82	4.7 ± 0.3	2.1 ± 0.2	**0.001**	2.1 ± 0.3	2.1 ± 0.23	0.56

**Table 7:** Changes of radiographic parameters preoperatively till the last follow-up in Groups R, S, and C (paired t-test).

**Group**	**Preop**	**Postop**	**Pre/Post**	**Postop-2**	**F-up **	**Postop-2/F-up**
		**6 mon**	**P-value**	**2 years**	**max**	**P-value**
	***Lordosis T12-S1(Cobb degrees)***					

**R**	48±11	43±8	0.15	43±7	44±8	**0.12**

**S**	47±13	49±11	0.49	48±11	48±13	0.64

**C**	39±16	41±13	0.58	40±12	42±12	0.32

	***SL (Cobb degrees)***					

**R**	18±8	23±6	**0.082**	22±6	23±6	0.78

**S**	22±12	23±10	0.67	23±9	23±10	0.49

**C**	22±16	26±8	0.27	27±6	26±9	0.65

	***SS (Cobb degrees)***					

**R**	31±8	34±7	**0.015**	33±8	34±6	0.30

**S**	33±10	31±7	0.46	30±6	31±7	0.82

**C**	27±10	31±10	**0.054**	30±12	31±10	0.63

	***PDH (Cobb degrees)***					

**R**	4±1	5±2	0.38	5±2	5±2	0.72

**S**	6±2	7±2	0.38	7±2	6±1	0.72

**C**	4±2	4±2	0.23	4±2	4±2	0.50

	***SVA (mm)***					

**R**	11±19	9±18	0.37	9±15	10±17	0.54

**S**	12±18	9±16	0.54	8±14	9±17	0.45

**C**	14±18	12±15	0.91	13±16	12±15	0.33

	***ADH (Cobb degrees)***					

**R**	6±2	7±2	0.34	7±3	6±2	**0.06**

**S**	10±2	12±1	0.34	11±3	11±2	0.15

**C**	7±3	7±2	0.71	6±3	6±2	**0.039**

	***PT (Cobb degrees)***					

**R**	33±2	25±10	0.52	24±11	26±9	**0.08**

**S**	23±7	22±9	0.17	23±9	24±8	0.17

**C**	28±7	29±6	0.18	28±7	30±6	0.73

**Table 8:** Demographic characteristics at baseline of 55 patients.

**Characteristics**	**Group R**	**Group S**	**Group C**	**P-Value**
**Number**	17	18	20	

**Female/male ratio**	(10:7)	(12:6)	(13:7)	0.72

**Age**	64±17	59±13	61±12	0.49

## Data Availability

The data used to support the findings of this study are available from the corresponding author upon request.
